# Supplementary material for: K-means quantization for a web-based open-source flow cytometry analysis platform
Source: Sci Rep. 2021 Mar 24;11:6735. doi: 10.1038/s41598-021-86015-6 (PMC7991430; doi:10.1038/s41598-021-86015-6)
Supplement: Supplementary file 1 — Supplementary Information. [file 41598_2021_86015_MOESM1_ESM.pdf]

Supplementary Figures

K-means Quantization for a Web-based Open-source Flow Cytometry Analysis Platform

*Nathan Wong[1], Daehwan Kim[1], Zachery Robinson[1], Connie Huang[1], *Irina M. Conboy[1]

[1]Department of Bioengineering and QB3, UC Berkeley, Berkeley, CA 94720, USA

***Correspondence to:**

Irina M. Conboy, **email:** iconboy@berkeley.edu

Nathan Wong, **email:** nathanwong@berkeley.edu

Supplemental Figures

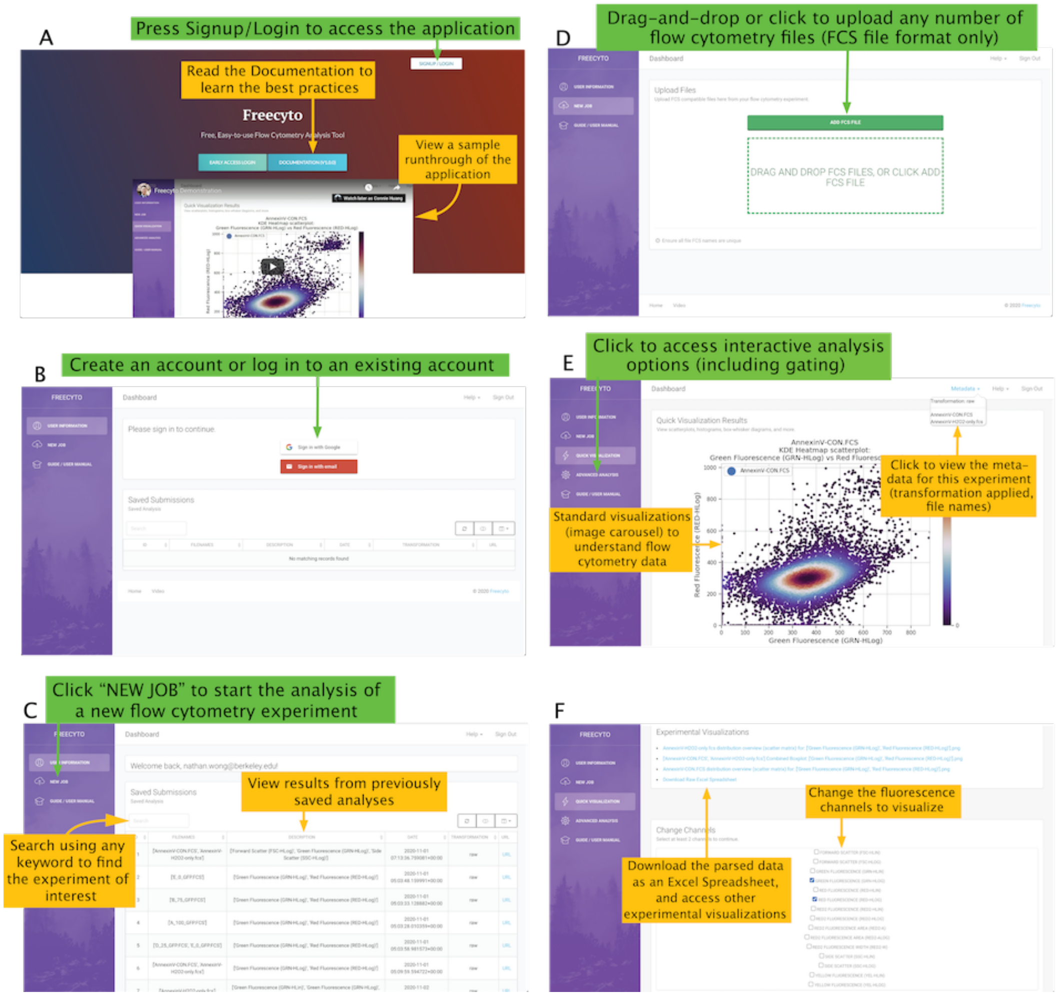

Supplemental Figure 1. Freecyto Quick Visualization Walkthrough.
(A) Freecyto Homepage. Navigate to freecyto.com and select login to continue. After clicking Login to access the Freecyto application interface, you need to create a new user account either through Google or email. If you already have an account on Freecyto, log in with those credentials.
(B) Freecyto Login Page. Create an account using a Google or Email ID. Once you have successfully logged in, you will be able to access your personal user portal. From here, you can see all past analyses that you performed (linked to your user ID). You can also sort and search past saved analyses and access visualizations of those analyses directly and quickly by clicking on the corresponding link.
(C) Freecyto User Portal. View previously performed analyses and access the page to create a new job. New users will have no previous experiments saved. However, each time the user uploads data or another user shares an experiment, the experiment will be listed in the table of the home page. These experiments can be sorted, indexed, and accessed without needing to repeat previously performed analysis operations. To begin a new job, click "New Job" located in the left column of the dashboard. Next, upload any number of FCS files you wish to analyze.
(D) Freecyto New Job. Upload new FCS file(s) to begin a new analysis job. After the files have been uploaded, you will be able to access the quick visualizations page, in which the standard histograms, scatterplots, heatmaps, and box-whisker diagrams are displayed in a slideshow (image carousel) format.
(E) Freecyto Quick Visualization. View histograms, scatterplots, box-whisker diagrams, heatmaps of the uploaded flow cytometry data. You may also change the fluorescence channels displayed at this time, by scrolling to the bottom of the page and selecting the new fluorescence channels to display.
(F) Changing the quick visualization display options.

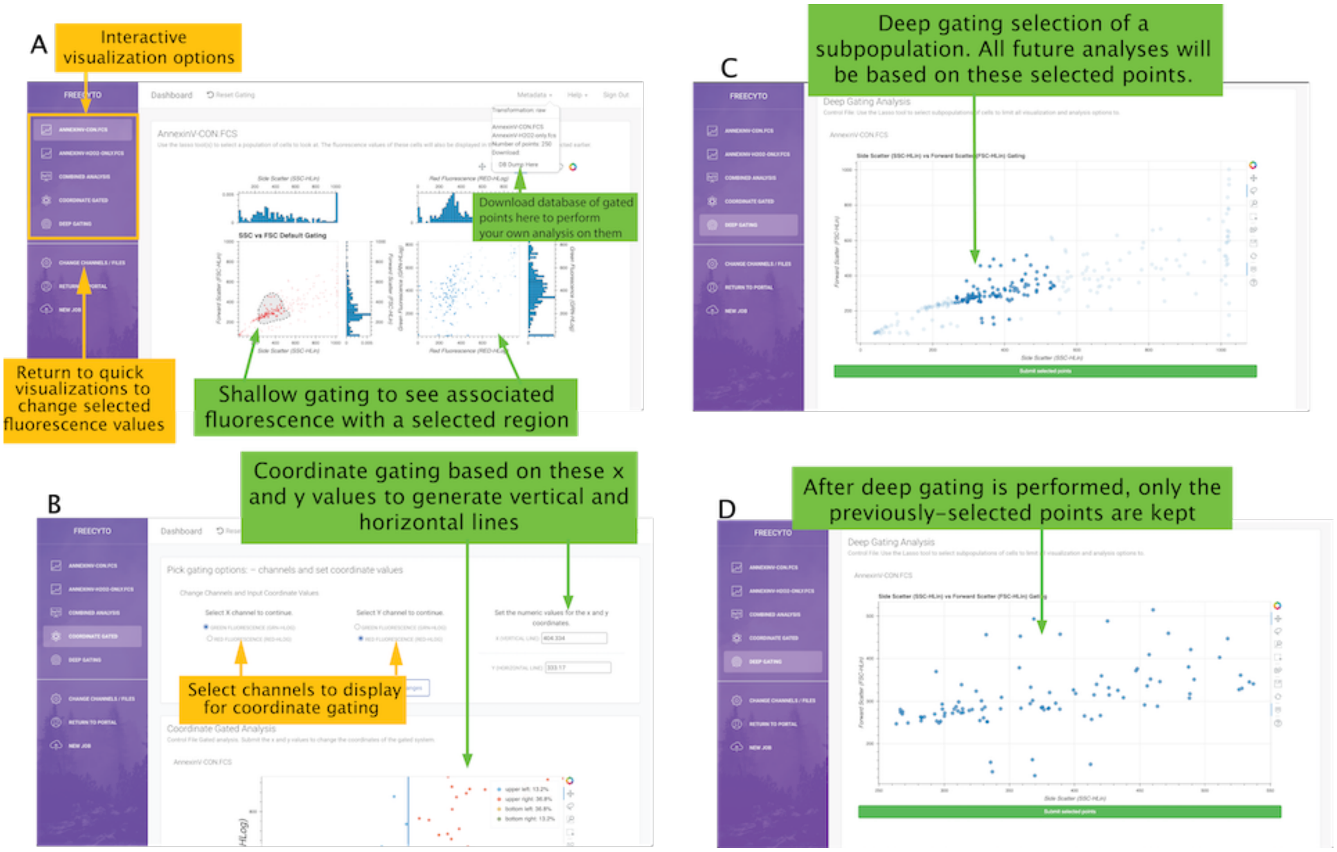

Supplemental Figure 2. Freecyto Interactive Analysis Walkthrough. Next, press "advanced analysis" to access the interactive visualizations of the flow cytometry data. This is an example of the shallow gating feature, in which selecting a sub-population of cells will display that sub-population across all selected fluorescence channels.
(A) Freecyto Interactive Shallow Gating. Shallow gating to see associated fluorescence values of a selected region. Coordinate gating analysis can then be performed to determine the percentage of cells that are located within or outside the bounds of preset x and y values.
(B) Freecyto Interactive Coordinate Gating Display. Gate flow cytometry experimental files based on specific X and Y values and see the percentage of cells within and outside these regions. Deep gating can also be performed to specifically examine sub-populations of cells.
(C) Freecyto Interactive Deep Gating Display (Before).
(D) Freecyto Interactive Deep Gating Display (After).

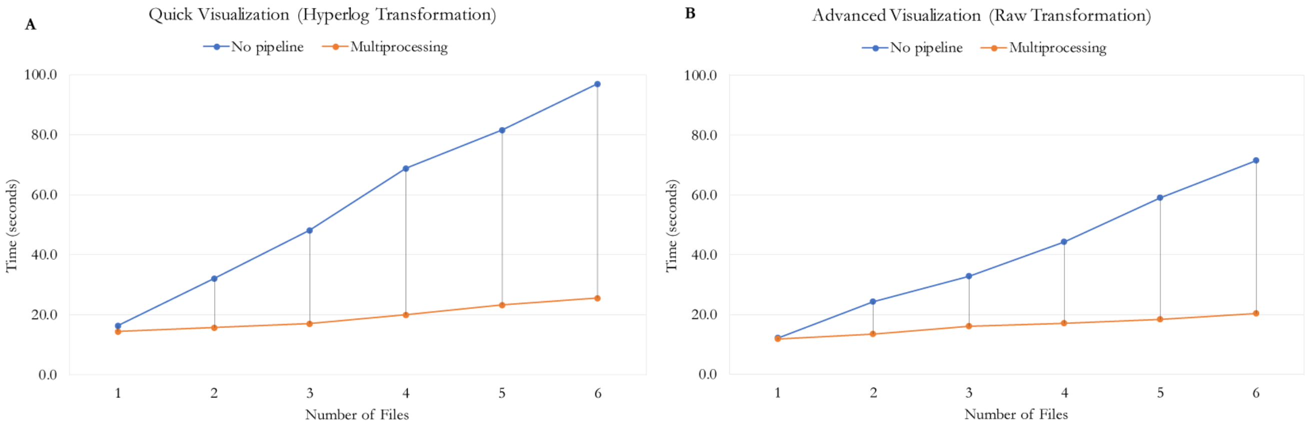

Supplemental Figure 3. Multiprocessing vs No Pipeline. Plots show the time taken to process files when using multiprocessing vs. no multiprocessing for **(A)** Quick visualization and for **(B)** Advanced visualization. Microsoft Excel[30] was used to generate supplemental figure 3.

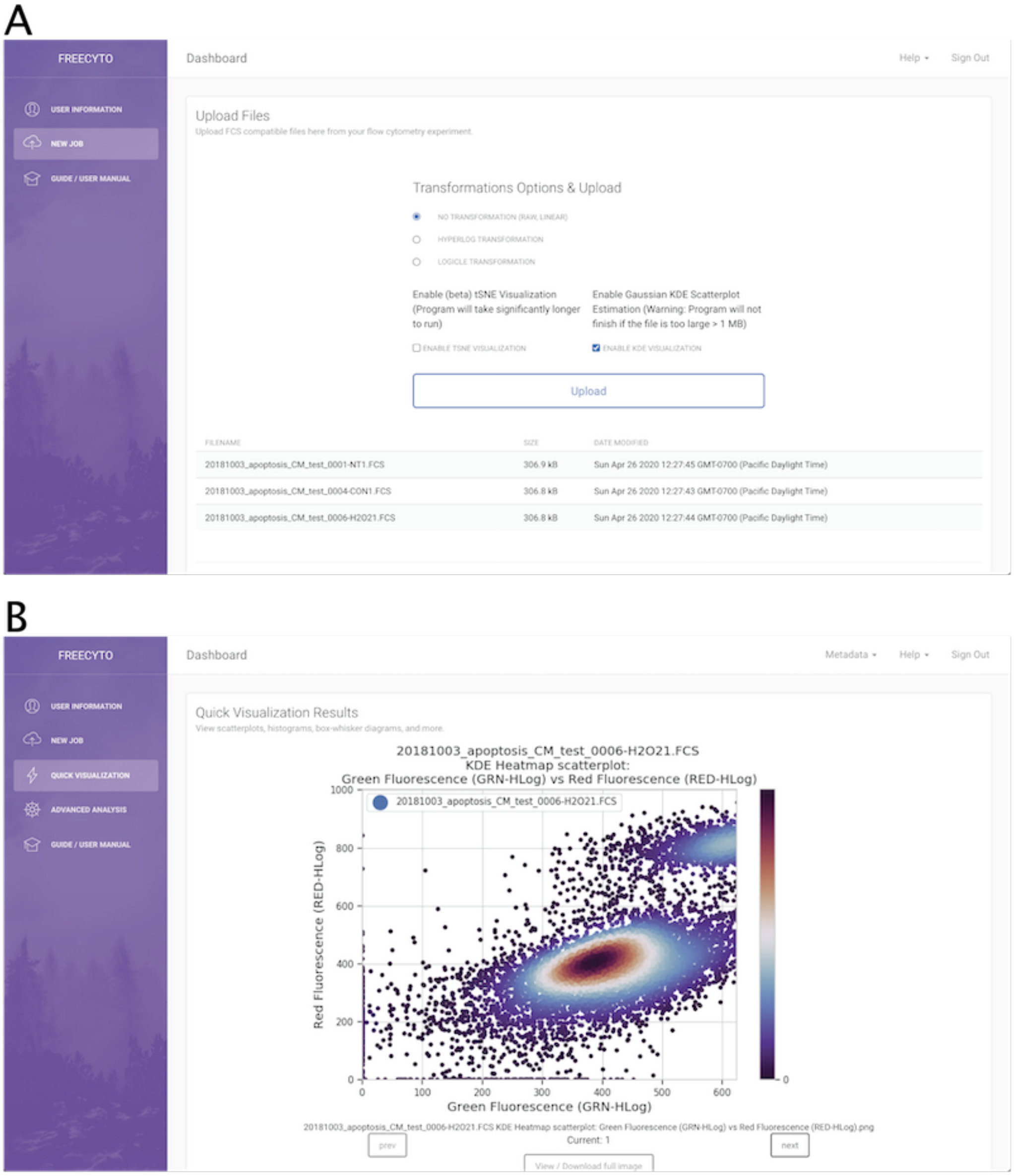

Supplemental Figure 4. Some advantages of the Freecyto analysis include multiple file upload and quick data visualization.
(A) Multiple File Upload. You can upload multiple files here and customize available settings, such as t-SNE and KDE visualizations with the option of various transformations.
(B) Quick Visualizations. You now have access to many different visualizations of your uploaded data, including histograms, kernel density plots, and heatmaps.

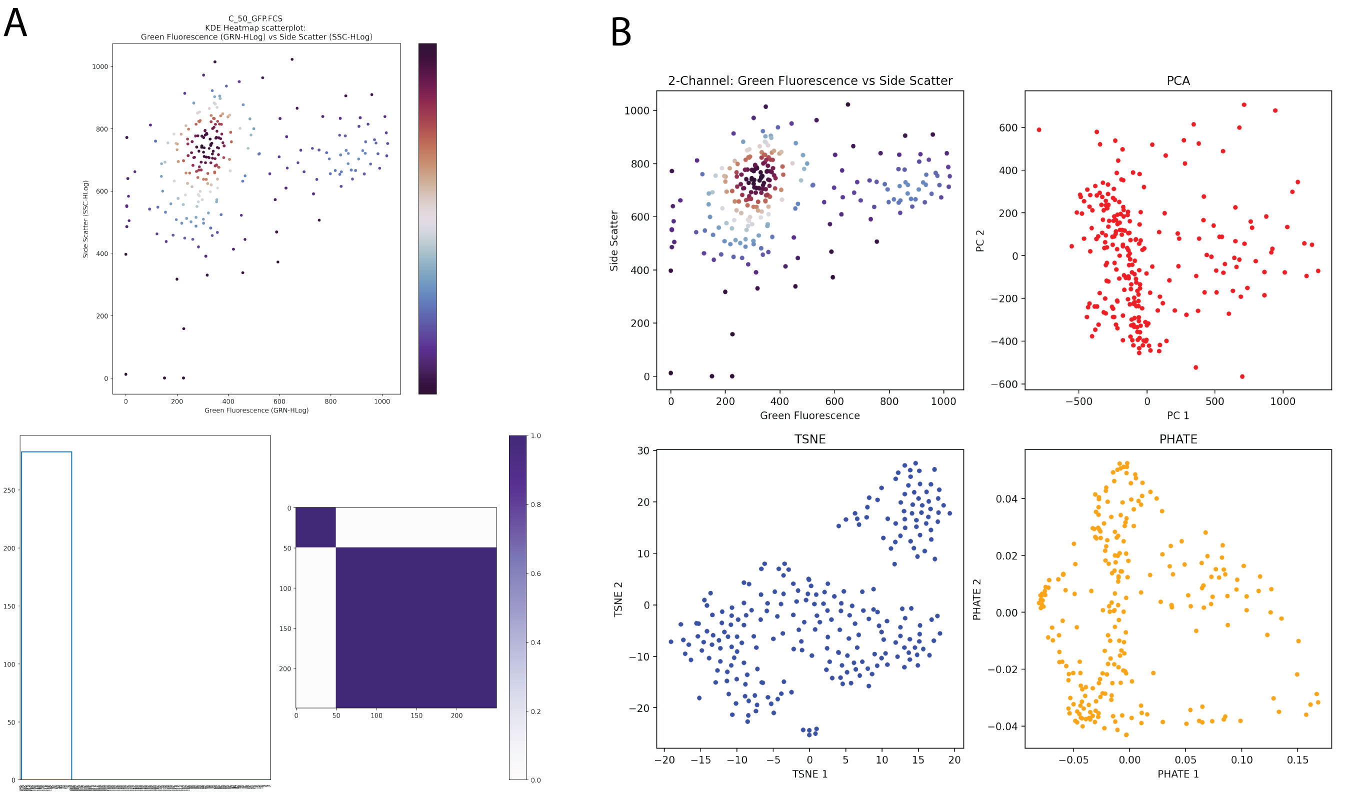

Supplemental Figure 5. Downstream analysis of flow cytometry experiments.
(A) Visualizing the local structure of the 50:50 WT/GFP+ experiment. Ward hierarchical clustering is performed downstream of the k-means quantization on the spearman correlation matrix of the Green Fluorescence and Side Scatter channels. We find the 2 distinct sub-populations as expected from this experiment.
(B) Dimensionality reduction comparison. Various dimensionality reduction techniques (PCA, tSNE, PHATE[33]) were performed on the same downstream data, but with all 15 channels selected as features. As expected, 2 distinct sub-populations were noted in each of these methods.
Note: Matplotlib[26] python library was used to generate supplemental figure 5.

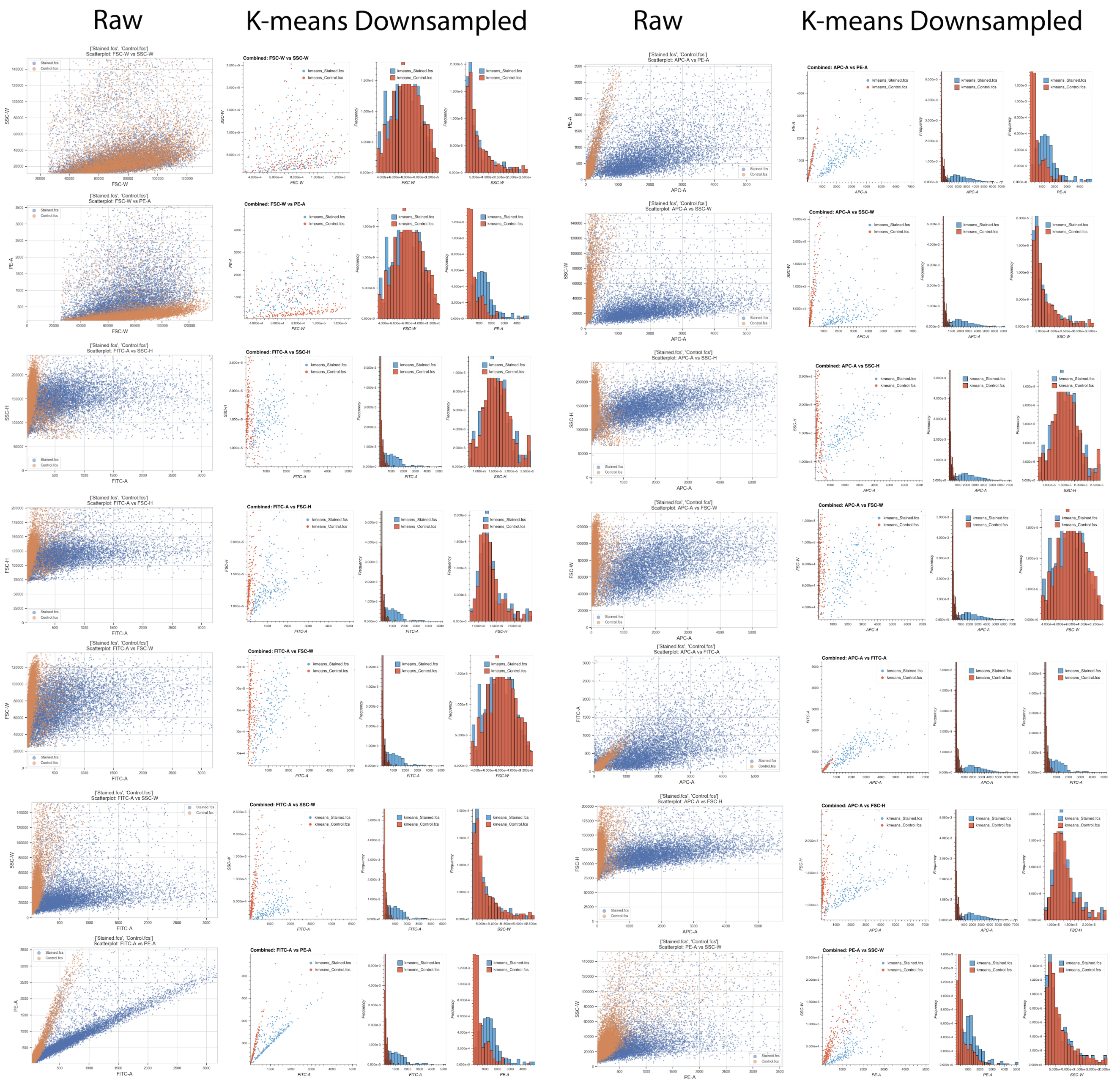

Supplemental Figure 6. Comparing k-means downsampling on multi-parameter dataset.
Raw analysis of the data is compared side-by-side against the k-means downsampled data (produced by Freecyto) on several color channels. The data used in this figure was retrieved from Flow Repository from experiment **RNA Flow Cytometry: K562 BCR mRNA expression**[34]. The accuracy of k-means downsampling is comparable to no downsampling in conserving data integrity.
Note: Matplotlib[26] python library was used to generate the raw data graphs in supplemental figure 6 and Bokeh[29] was used to generate the K-means downsampled data graphs.
